# Supplementary material for: The political consequences of opioid overdoses
Source: PLoS One. 2020 Aug 4;15(8):e0236815. doi: 10.1371/journal.pone.0236815 (PMC7402477; doi:10.1371/journal.pone.0236815)
Supplement: S1 Text — (PDF) [file pone.0236815.s001.pdf]

1 Aggregate estimates

Assuming, based on our data from Connecticut, that each overdose victim has on average five friends or family, we estimate two million total friends and family affected by the opioid epidemic since 1999. If they vote at a rate 10 percentage points lower than the statewide average as per Figure 1, we expect 200,000 fewer votes in the 2016 General Election. While this figure is not large enough to swing any individual state when these victims are allocated nationally, many state and local races are well within this margin.

Secondly, if these friends and family defect join the Democratic party at a rate of 2 percentage points higher than the statewide average, we expect an additional 40,000 Democratic registrants (and consequently, an equal number fewer Republican registrants).
